# Supplementary material for: Antropo: An open-source platform to increase the anthropomorphism of the Franka Emika collaborative robot arm
Source: PLoS One. 2023 Oct 18;18(10):e0292078. doi: 10.1371/journal.pone.0292078 (PMC10584189; doi:10.1371/journal.pone.0292078)
Supplement: S1 File — For design files, bill of materials, required tools, build instructions, and operation instructions. (PDF) [file pone.0292078.s001.pdf]

Antropo: An open-source platform to increase the anthropomorphism of the Franka Emika robot
USER'S GUIDE

Constantin Scholz[1,2], Hoang-Long Cao[1,3], Ilias El Makrini[1,3], Bram Vanderborght[1,2]

[1]Brubotics, Vrije Universiteit Brussel, Pleinlaan 2, Brussels 1050, Belgium
[2]IMEC, Kapeldreef 75, Leuven 3001, Belgium
[3]Flanders Make, Vrije Universiteit Brussel, Pleinlaan 2, Brussels 1050, Belgium

constantin.florian.scholz@vub.be — twitter: cfscholz
hoang.long.cao@vub.be — twitter: HoangLongCao
ilias.el.makrini@vub.be
bram.vanderborght@vub.be — twitter: BramVDBorght

1. Design files

This section contains a summary of the design files used to build the elements making up the platform. All files can be found at their respective links in the online repositories presented in Table 1. It is recommended to utilise the **CAD_FULL_Source** as a starting point for design familiarisation. Description for each design file presented in Table 1 is as follows.

Design Filename	File type	License	Location of the file
CAD_FULL_Source	F3Z	CC BY 4.0	https://doi.org/10.17632/9wnd37wv7c.1
STEP_D1_Diffuser	STEP	CC BY 4.0	https://3dprint.nih.gov/discover/3DPX-017000<br>https://doi.org/10.17632/9wnd37wv7c.1
STEP_D2_DiffuserHolder	STEP	CC BY 4.0	https://3dprint.nih.gov/discover/3DPX-017001<br>https://doi.org/10.17632/9wnd37wv7c.1
STEP_D5_DiffuserBatteryHolder	STEP	CC BY 4.0	https://3dprint.nih.gov/discover/3DPX-017002<br>https://doi.org/10.17632/9wnd37wv7c.1
STEP_C1_Camera	STEP	CC BY 4.0	https://3dprint.nih.gov/discover/3dpx-016999<br>https://doi.org/10.17632/9wnd37wv7c.1
STL1_D1_Diffuser	STL	CC BY 4.0	https://3dprint.nih.gov/discover/3DPX-017000<br>https://doi.org/10.17632/9wnd37wv7c.1
STL2_D2_DiffuserHolder	STL	CC BY 4.0	https://3dprint.nih.gov/discover/3DPX-017001<br>https://doi.org/10.17632/9wnd37wv7c.1
STL3_D5_DiffuserBatteryHolder	STL	CC BY 4.0	https://3dprint.nih.gov/discover/3DPX-017002<br>https://doi.org/10.17632/9wnd37wv7c.1
STL4_C1_Camera	STL	CC BY 4.0	https://3dprint.nih.gov/discover/3dpx-016999<br>https://doi.org/10.17632/9wnd37wv7c.1
DXF1_D3_DiffuserSidePanelLeft	DXF	CC BY 4.0	https://doi.org/10.17632/9wnd37wv7c.1
DXF2_D4_DiffuserSidePanelRight	DXF	CC BY 4.0	https://doi.org/10.17632/9wnd37wv7c.1
DXF3_C2_CameraLens	DXF	CC BY 4.0	https://doi.org/10.17632/9wnd37wv7c.1
FORM_D1_Diffuser	FORM	CC BY 4.0	https://3dprint.nih.gov/discover/3DPX-017000<br>https://doi.org/10.17632/9wnd37wv7c.1
GCODE1_D2_DiffuserHolder	GCODE	CC BY 4.0	https://3dprint.nih.gov/discover/3DPX-017001<br>https://doi.org/10.17632/9wnd37wv7c.1
GCODE2_D5_DiffuserBatteryHolder	GCODE	CC BY 4.0	https://3dprint.nih.gov/discover/3DPX-017002<br>https://doi.org/10.17632/9wnd37wv7c.1
GCODE3_C1_Camera	GCODE	CC BY 4.0	https://3dprint.nih.gov/discover/3dpx-01699<br>https://doi.org/10.17632/9wnd37wv7c.1
PDF_D6_DiffuserElectronics	PDF	CC BY 4.0	https://doi.org/10.17632/9wnd37wv7c.1
INO_CX1_WebModule	INO	MIT	https://doi.org/10.17632/9wnd37wv7c.1
INO_CX2_RosModule	CPP	MIT	https://doi.org/10.17632/9wnd37wv7c.1
ZIP_TUN_SoundRepository	ZIP	CC BY 4.0	https://doi.org/10.17632/9wnd37wv7c.1

Table 1: List of all project design files required to build Antropo.

CAD_Full_Source: Full repository for the combined hardware elements installed on the cobot. The file can be opened and edited with Autodesk Fusion 360[1]. If required the Franka Emika Cobot Panda CAD can be downloaded from WiredWorkers or Franka Emika, it is not included in the repository [7, 1].

STEP_D1_Diffuser: Individual standard for the exchange of product Data CAD file for translucent LED diffuser of light module.

[1]https://perma.cc/57MM-D3NK

STEP_D2_DiffuserHolder: Individual standard for the exchange of product Data CAD file for mounting light module on Franka Emika Panda.

STEP_D5_DiffuserBatteryHolder: Individual standard for the exchange of product data CAD file to hold the battery and mount it on the light module.

STEP_C1_Camera: Individual standard for the exchange of product data CAD file for camera base of camera module.

STL1_D1_Diffuser: Individual standard triangle language CAD file for translucent LED diffuser of light module.

STL2_D2_DiffuserHolder: Individual standard triangle language CAD file for mounting light module on Franka Emika Panda.

STL3_D5_DiffuserBatteryHolderr: Individual standard triangle language CAD file to hold the battery and mount it on the light module.

STL4_C1_Camera: Individual standard triangle language CAD file for camera base of camera module.

DXF1_D3_DiffuserSidePanelLeft: Drawing exchange format file for laser cutting left side panel of light module.

DXF2_D4_DiffuserSidePanelRight: Drawing exchange format file for laser cutting right side panel of light module.

DXF3_C2_CameraLens: Drawing exchange format file for laser cutting camera lens of camera module.

FORM_D1_Diffuser: Formlabs Form 2[2] resin 3D printer instruction file to print diffuser of light module.

GCODE1_D2_DiffuserHolder: Prusa Mini+ 3D[3] printer gcode printing instructions to print holder of light module for Franka Emika Panda.

GCODE2_D5_DiffuserBatteryHolder: Prusa Mini+ 3D gcode printing instructions to print battery holder of light module.

GCODE3_C1_Camera: Prusa Mini+ 3D gcode printing instructions to print camera base of camera module.

INO_CX1_WebModule: Code for Arduino UNO WiFi Rev 2 to set, control, manipulate the light and sound and host a Wifi connection hotspot and web interface.

INO_CX2_RosModule: Code for Arduino UNO WiFi Rev 2 to set, control, manipulate the light and sound and host a ROS connection as a subscriber.

ZIP_TUN_SoundRepository: This file is the full zip repository of three proposed open-source mp3 sound files that can be used in human-robot-interaction research.

[2]https://perma.cc/99ZW-YFTM
[3]https://perma.cc/T3RB-3YW7

2. Bill of materials

The bill of materials (BOM) presented in Table 2 lists the required components and their description, component identifiers in the CAD drawings, quantity needed, total cost, supplier links, and material type. The cost of the components excludes VAT and is based on pricing for shipment to Belgium. The pricing is based on the cost per exact amount of components needed to build Antropo and not the minimum order amount from the supplier. The rationale behind this is twofold. Firstly supply prices and minimum order quantities vary worldwide, and a large proportion of the components required might be already available in stock in the design facilities for building Antropo. We have chosen RS Components[4] which ships internationally, as the main supplier. Apart from three components, everything can be directly procured via RS. To allow for fast, international procurement based on current available worldwide stock, we provide a BOM on Octopart[5], which optimises the procurement and finds availability of components, also if they should be discontinued at RS. The tool also allows querying the pricing in any currency worldwide. Apart from this, we made all component links available in Perma.cc[6] to prevent link death and provide a BOM as a PDF, Excel and CSV.

[4] https://perma.cc/LTJ3-QEJQ
[5] https://perma.cc/BX9Z-N3Q2
[6] https://perma.cc/

Component Identifier	Component Description	Quan.	Cost/ Quan. - €	Total Cost - €	Supplier Link perma.cc/...	Material Type
SCR_1_M3<br>SCR_2_M3	M3x25mm, Countersunk Steel Screw	2	0.046	0.09	XFD7-SXSE	Metal
NUT_1_M3<br>NUT_2_M3	M3 Lock Nut	2	0.052	0.10	RPM3-GAXB	Metal
WAS_1_M3<br>WAS_2_M3	M3 Washer	2	0.006	0.01	F3SN-KXMJ	Metal
SCR_3_M2<br>SCR_4_M2	M2x8mm, Pozidriv Screw	2	0.036	0.07	2NEU-RKXW	Metal
D4_DiffuserSidePanelRight<br>D3_DiffuserSidePanelLeft<br>C2_CameraLens	Clear PlexiGlas Sheet	70 cm^2	0.01	0.70	HMG5-PE5Z	Polymer
C1_Camera	Black PLA Filament, 1.75mm	23g	0.046	1.06	7WZJ-973Q	Polymer
D2_DiffuserHolder<br>D5_DiffuserBatteryHolder	Light Grey PLA Filament, 1.75mm	104g	0.036	3.75	S9BP-V4J7	Polymer
D1_Diffuser	FormLabs Clear Resin	101ml	0.11	10.94	2LKK-GDTE	Polymer
A5_BatteryAdhesive<br>A2_LEDAdhesive<br>A3_ArduinoAdhesive	Adhesive Foam Tape, 9mm Wide, 0.8mm Thick	55cm	0.057	3.14	53PS-3EVD	Polymer
T3_ArduinoUnoWifiRev2<br>T2_ArduinoHolder	Arduino, UNO WiFi Rev 2 + Holder	1	40.36	40.36	G4PD-GSNQ	Semicond.
T4_PowerBank	PowerBank, 5V, 2.1A USB-A 5000mAh	1	16.8	16.80	AP4D-LN5D	Semicond.
T1_LEDStrip	Adafruit DotStar RGB LED Strip, 144 LED/m	1	42.74	42.74	XY5X-L7DM	Semicond.
T6_ScrewTerminal	3-pin Screw Terminal	2	0.88	1.76	RL6N-EWZX	Composite
T5_ProtoShield	Arduino, Proto Shield Rev3	1	9.66	9.66	T2PM-GU6M	Composite
T7_Connector	USB Connector to power	1	2.52	2.52	FHP7-AZRK	Composite
T8_Buzzer	Piezo Buzzer	1	2.52	2.52	8JBE-CCWY	Composite
T9_Transistor	Bipolar NPN Transistor	1	0.20	0.20	R6LF-WRUW	Composite
CD1_Cable	0.5 mm^2 Equipment Wire, 16/0.2 mm	10cm	0.002	0.02	L7QG-ZA8P	Composite

Table 2: Bill Of Materials of all components required to build Antropo. A PDF, Excel, CSV, and internationally procurable BOM via Octopart can be found in the Project Repository in the BOM folder.

3. Required Tools

Table 3 presents the tools we utilised to build Antropo as described in section 4. To continue with section six, it is essential to have the following or similar tools available.

Tool Identifier	Tool Name	Link to Tool perma.cc/...
T1	Franka Emika Panda Cobot	3KQN-D2KQ
T2	Form Labs 2 3D Printer	99ZW-YFTM
T3	Prusa Mini+ 3D Printer	T3RB-3YW7
T4	LTT iLaser 4000 Laser Cutter	MW3C-SDG7
T5	Nitril Gloves	C6KB-QDGT
T6	Form Wash	2L98-CKH6
T7	Form Labs Cure UV	2L98-CKH6
T8	Form Labs Resin Tank	N69M-7DLK
T9	Side Cutter	7CV2-6MWU
T10	Pozidriv M2 Screw Driver	AVV5-ANGL
T11	Philips M3 Screw Driver	AVV5-ANGL
T12	Spanner Set	ALV6-D3P3
T13	Scissors	CUK6-R7AK
T14	Wire stripper	N2VX-NQAF
T15	Soldering Iron	HM33-8EK4
T16	PCB Holder	QW96-FQRE
T17	Dremel + Mill Kit	XP9G-JK3P
T18	USB-B to USB-A Cable	4X2P-7EXY
T19	USB Stick	BX8K-BSZX
T20	Solder	4JNG-ZH87
T21	Super Glue	3ZMT-UPCW
T22	Hex Key Size 4	57JQ-UGVE

Table 3: Tools required to build Antropo.

4. Build instructions

The following section guides through the different steps of building Antropo. The installation of the modules onto the Franka Emika Panda Cobot, the programming of the light and sound module will be explained in section 5.

4.1 Camera Module

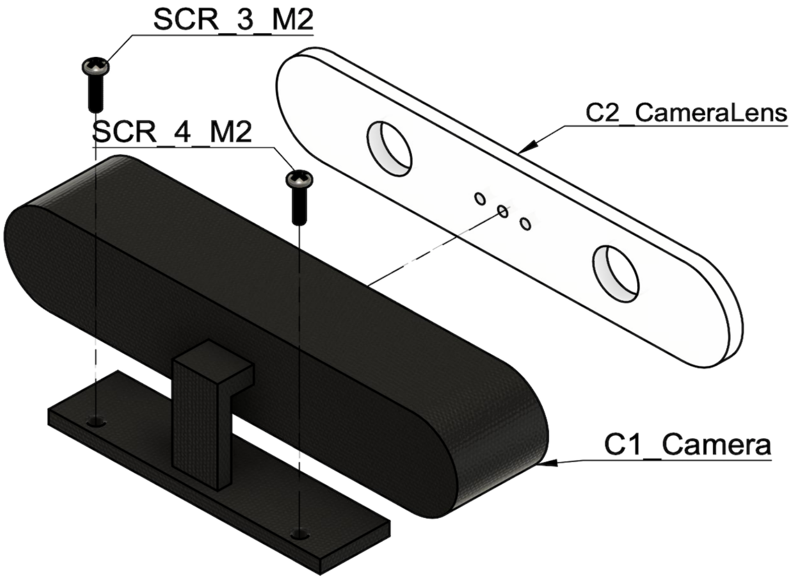

Figure 1: Exploded Assembly Drawing of Camera Module.

Camera Module assembly steps:

1. **3D Print C1_Camera**
   - 1.1 Change the filament on the Prusa Mini+ 3D Printer (T3) to black PLA and transfer the file GCODE3_C1_Camera to a USB stick (T4).
   - 1.2 For best print results, start a first layer calibration on the Prusa Mini+ printer [5].
   - 1.3 Start the print of the camera; it will run for around 2 hours and 20 minutes with the gcode provided.
   - 1.4 Once the print is completed, remove it from the base plate of the printer and remove the excess support material with a side cutter (T9).
   - 1.5 Make sure the support from the screw holes on the base is fully removed.
2. **Laser cut C2_CameraLens**
   - 2.1 Load the DXF3_C2_CameraLens into your laser cutter software. We are utilising the software LightBurn[7] for this purpose. Set all visible lines of the inserted DXF to cut at 10-15mm/s at 90% power output. Upload the settings to the laser cutter (T4).

[7] https://perma.cc/3HNU-JPP3

2.2 Insert the clear PlexiGlas sheet into the laser cuter.

2.3 Close the lid of the laser cutter and set the origin to the top right corner, frame the design and start the air extraction of the laser cutter.

2.4 When the extraction is running, start the cutting process. Once the process is completed, remove the camera lens and put the PlexiGlas sheet aside for building the light module later.

3. **Combining the Camera Module elements**

3.1 Take the superglue (T21) and put two small spots on the outer circumference of the printed C1_Camera.

3.2 Glue the C2_CameraLens as seen in Figure 1 onto the C1_Camera base.

3.3 Insert the two M2 Pozidriv screws through the base. The screws will later be screwed onto the Franka Hand end effector.

4.2 **Light Module**

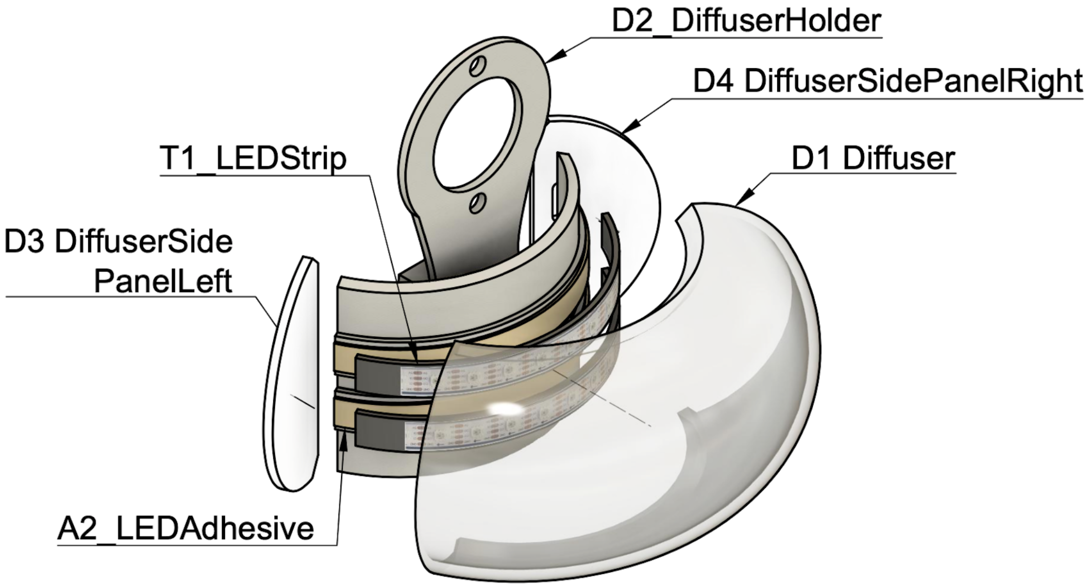

Figure 2: Exploded Assembly Drawing of Diffuser, Holder and LED Strip of Light Module.

Light Module assembly steps:

1. **3D print Diffuser Holder, Diffuser, cut Side Panels and install the LED strip.**

1.1 As described in the Camera Module assembly section, follow the same process to 3D print the D2_DiffuserHolder with the Prusa Mini+ 3D Printer (T3) in light grey PLA. Utilise the file GCODE1_D2_DiffuserHolder. The print will last for about 9 hours and 30 minutes.

1.2 Change the resin of the Form Labs 2 3D Printer (T2) to clear resin. If needed, insert a new resin tank (T8). Wear nitrile gloves (T5) and make sure the base plate of the printer is clean before continuing to the next step.

1.3 On your computer, open the PreForm[8] software. Insert the FORM_D1_Diffuser file, connect the computer to the printer and start the print. The print of the D1_Diffuser will last around 11 hours and 30 minutes.

1.4 Following the steps in the Camera Module assembly section cut the D4_DiffuserSidePanelRight and D3_DiffuserSidePanelLeft with the related DXF files.

1.5 Take the Adhesive Foam Tape and cut two 13cm long strips for the A2_LEDAdhesive.

1.6 Unpack the Adafruit DotStar T1_LEDStrip. The strip comes with wires soldered onto one of the ends. From this end count of 28 LEDs, cut off the rest of the LEDs behind the 28th LED along the cutting mark.

1.7 We want to place two strands with each 14 LEDs under the Diffuser. Count 14 LEDs from the side where the cables are attached and cut the stripe along the cutting mark. You should now have two strips. One stripe with cables and 14 LEDs and a second stripe with only 14 LEDs.

1.8 Once the print of part D2_DiffuserHolder is completed, remove the support material. Attach the two A2_LEDAdhesive strips as shown in Figure 2.

1.9 Insert the cables of the T1_LEDStrip through the hole in the D4_DiffuserSidePanelRight. With the upper A2_LEDAdhesive, glue the LED strip onto the D2_DiffuserHolder.

1.10 Next, glue the 2nd strip of the T1_LEDStrip onto the lower adhesive. Cut four 2cm long wires (CD1_Cable) and solder (T20) (T15) the cables to the solder pads connecting CLOCK, DATA, VCC and GND of the two strips together. The two strands should now be connected to each other.

1.11 Once completed, remove the D1_Diffuser 3D print from the printer wearing Nitril gloves (T5). Place the base plate with the print in the Form Wash (T6) for 15 minutes.

1.12 With the side cutter (T9), remove all support material close to the surface of the D1_Diffuser if required, smooth the surface with the Dremel (T17).

1.13 Place the print in the Form Labs Cure UV (T7) for one cycle.

1.14 Spread super glue (T21) onto the edges of the D1_Diffuser and D2_DiffuserHolder and glue them together.

1.15 Glue the side panels D4_DiffuserSidePanelRight and D3_DiffuserSidePanelLeft onto the sides with super glue (T21). Let everything dry for 10 minutes.

2. **Attaching D5_DiffuserBatteryHolder to D2_DiffuserHolder**

[8] https://perma.cc/NS5U-5JFU

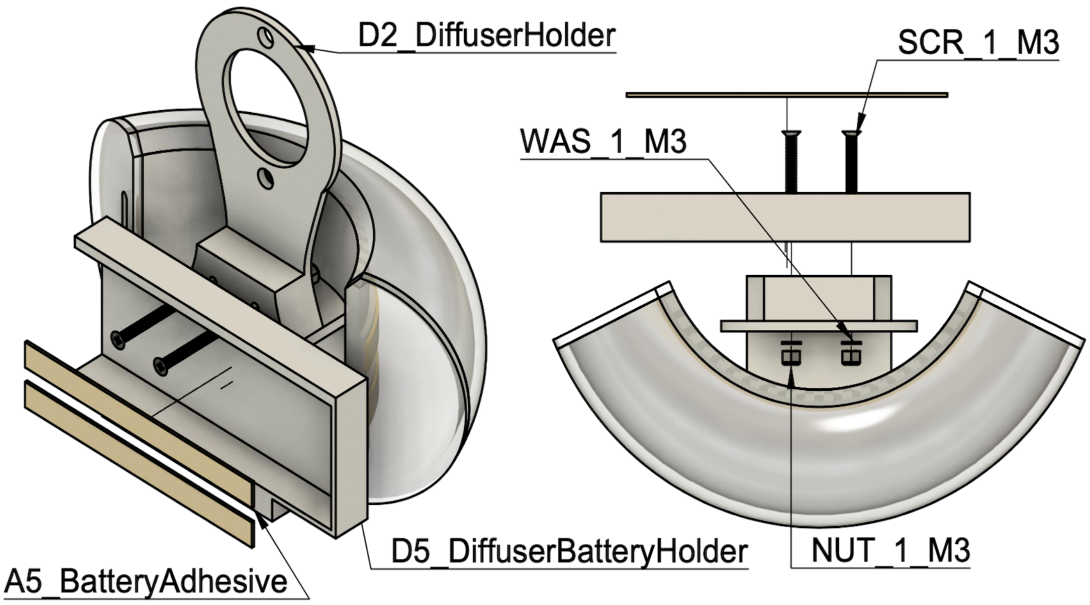

Figure 3: Exploded Assembly Drawing Battery Holder.

2.1 3D print the D5_DiffuserBatteryHolder with the Prusa Mini+ 3D Printer (T3) in light grey PLA. Utilise the file GCODE2_D5_DiffuserBatteryHolder. The print will last for about 2 hours and 15 minutes.

2.2 For the A5_BatteryAdhesive, cut 2 strips of foam tape, each 10cm long.

2.3 Once the 3D print is completed, remove the support material.

2.4 With the two M3 screws, nuts and washers, attach the D5_DiffuserBatteryHolder to the D2_DiffuserHolder utilising the M3 screwdriver (T11) and a spanner (T12). Connect both parts tightly together.

2.5 Glue the two strips of A5_BatteryAdhesive into the inside of the D5_DiffuserBatteryHolder as seen in Figure 3. Keep the tape cover facing you on the tape.

3. **Installing the Electronics**

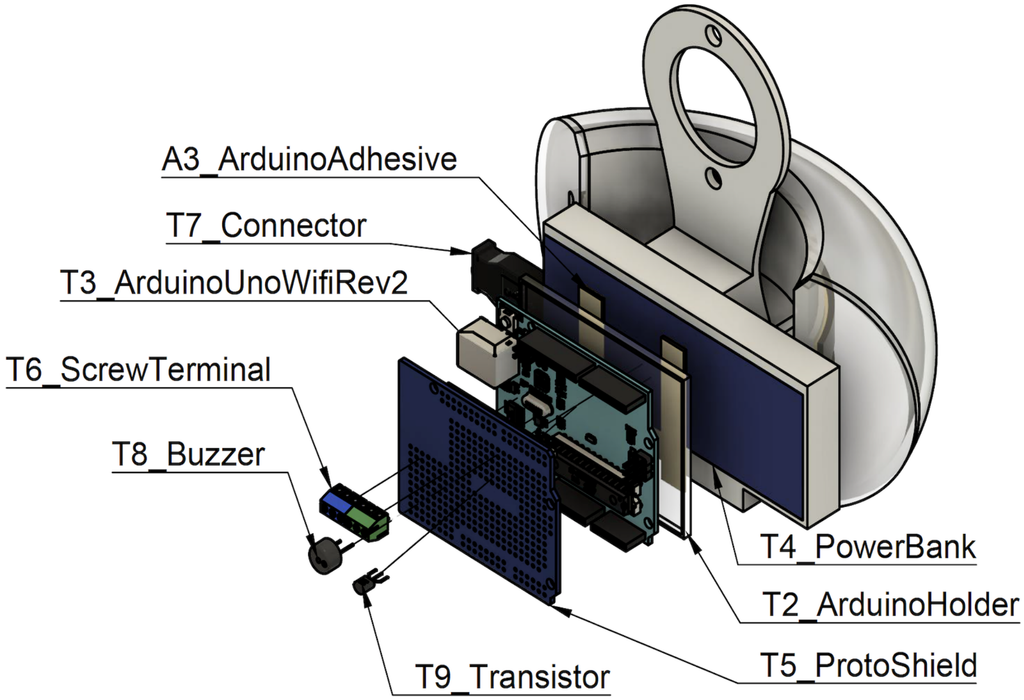

Figure 4: Exploded Assembly Drawing of Control Electronics of Light Module.

3.1 Remove the tape cover of the A5_BatteryAdhesive and glue the T4_PowerBank into the battery holder with the USB-A port of the power bank in the bottom left, as seen in Figure 4.

3.2 For the A3_ArduinoAdhesive cut 2 strips of the foam tape, each 5cm long.

3.3 Glue the T2_ArduinoHolder with the adhesive onto the battery and battery holder. The Arduino holder ships with the original Arduino Uno Wifi Rev 2.

3.4 Click the T2_ArduinoUnoWifiRev2 [4] into the T2_ArduinoHolder.

3.5 Solder the pin headers that are shipped with the T5_ProtoShield [3] onto the proto shield. Next, solder the two T6_ScrewTerminal [9], T8_Buzzer [8] and T9_Transistor [11], onto the bottom left half of the shield with the input holes facing downwards.

3.6 Solder connections from screw terminals to the Arduino pins. Use schematic PDF_D6_DiffuserElectronics or Figure 5.

3.7 Screw the cables of the T1_LEDStrip and the T7_Connector [12] cable ends tightly into the T6_ScrewTerminals.

3.8 Push the T7_Connector into the power bank.

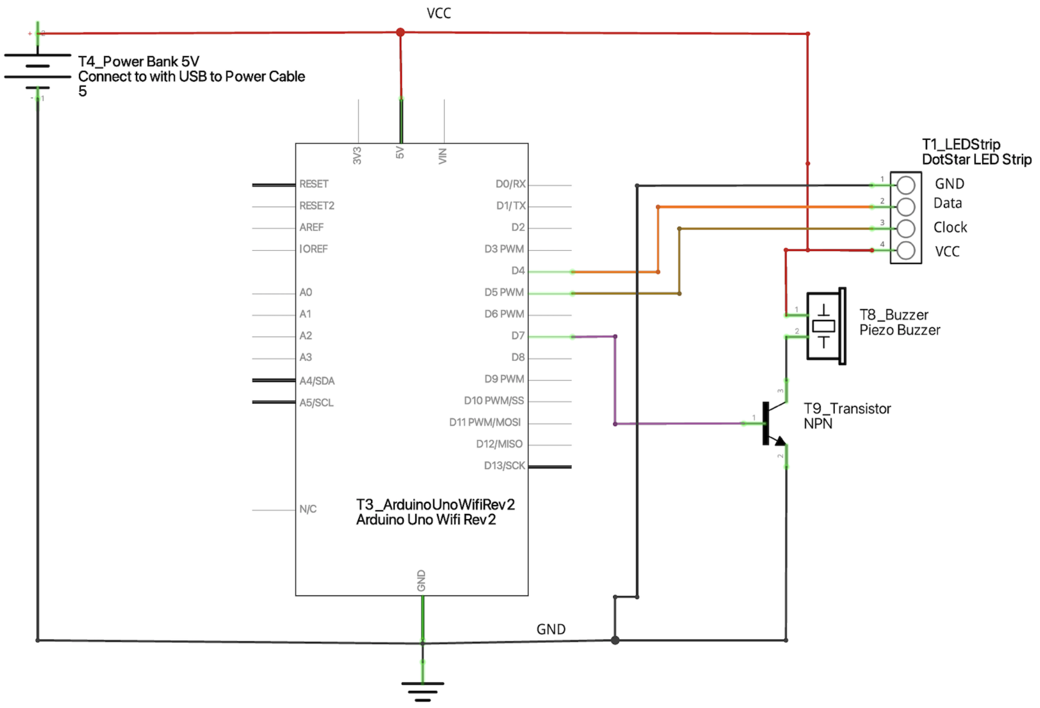

Figure 5: Schematic of Antropo electronics. Two LED strips and a piezoelectric buzzer are connected to an Arduino UNO Wifi Rev2, powered by a 5V power bank.

5. Operation instructions

5.1 Programming the Light and Sound Module

The light and sound module can either be controlled by connecting to it over Wifi from any browser or by connecting to it via ROS with a wired USB serial connection. We have programmed the module to display the colours presented in section **??**, the code is well commented and can be rapidly modified to match the desired colour animations. The module is using the Adafruit DotStar [2] and Arduino SPI [10] library, see the library examples for further reference. When utilising it via the Wifi connection the Wifi Nina Library [13] is used and for ROS the ROS Serial Arduino Library [6] is used. The next steps will guide you through installing the software onto the light module and playing back the colours and sounds.

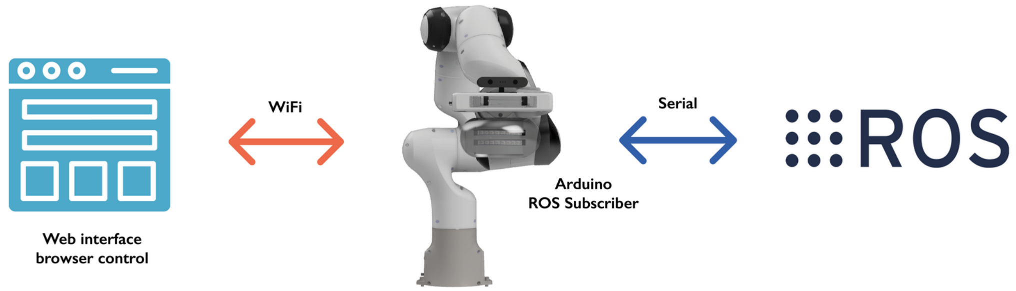

Figure 6: The light and sound module can be either interfaced via a web browser over Wifi or via ROS serial as a subscriber.

Connect to Light and Sound Module with Wifi and a Browser

1. On your computer, install the Arduino IDE[9].
2. Connect your Arduino Uno Wifi Rev2 to your computer with the USB cable. In the board manager of the IDE, select the board. Make sure that it appears in the port menu of the IDE.
3. Create a new file and copy over the contents from INO_CX1_WebModule into the new file.
4. Press the upload button and load the script onto the Arduino. If needed, install the missing libraries such as WifiNina and Adafruit_DotStar and SPI.
5. Once the upload is completed, the Arduino will advertise a Wifi network with the SSID **FRANKA_LIGHT**.
6. With your computer connect to the Wifi network, enter the password **franka123**.
7. Go into any browser, enter the following IP **http://192.168.4.1** and connect to it.
8. You will see an interface with 9 buttons allowing you to trigger and play the breathing, handover, action correct and action incorrect light animation in their respective colours as defined in section **??**, you can also switch the light module off as well as play the matching sounds.

Connect to Light Module with ROS

[9] https://perma.cc/56PH-WS8S

1. We assume that ROS is installed on your ubuntu machine.
2. Connect your Arduino Uno Wifi Rev2 to your computer with the USB cable. In the board manager of the IDE select the board. Make sure that it appears in the port menu of the IDE. Identify the port name the Arduino is connected to. You can identify this by running **ls dev/tty***.
3. Create a new file in the Arduino IDE and copy over the contents from INO_CX1_RosModule into this file.
4. Press the upload button and load the script onto the Arduino. If needed, install the missing libraries such as SPI and ROS Serial Arduino.
5. The Arduino is set up as a subscriber node that reacts to input commands in the form of integers and triggers the behaviour in form of light and sound. The commands triggering the behaviours can be found in table 4.
6. Open a new terminal window and launch **roscore**.
7. To interface the serial connection correctly with roscore enter: **rosrun rosserial_python serial_node.py _dev_ttyXXXX** in a new terminal window. Note: replace ttyXXXX with the correct serial port as identified in step 2.
8. To send commands to the Arduino that acts as a subscriber, enter the following command in a new terminal window, i.e to launch the breathing **rostopic pub antropo std_msgs_uint16 1**.

**Integer Triggers (UInt)**	**Behaviour**
1	Breathing animation
2	Green
3	Red
4	Blue
5	Light and sound off
6	Handover sound
7	Correct sound
8	Incorrect sound

Table 4: ROS UInt Light and sound module behaviour triggers.

5.2 Installation of the Light, Sound and Camera Module onto the Cobot

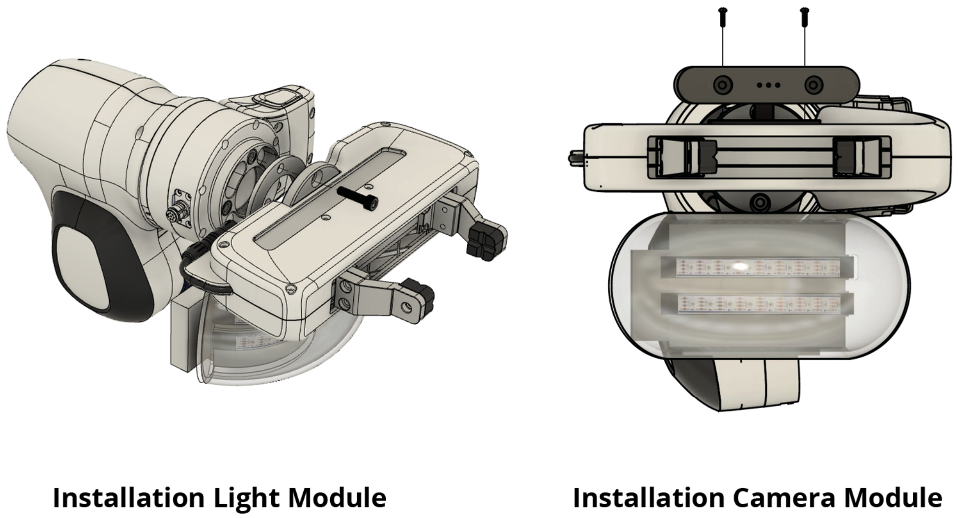

Figure 7: Installation of modules onto cobot.

1. Power off the cobot.
2. Unplug the end effector known as Franka Hand.
3. Utilise the hex key in size 4 (T22) and unscrew the two bolts that fix the end effector onto the robot arm. Make sure to hold onto the Franka Hand so that it does not fall down.
4. With a person assisting you place the Light Module D2_DiffuserHolder in between the Franka Hand and the tool-head. Together with your assistant, screw the two bolts through the holes of the end effector, Light Module and back into the tool-head as seen in Figure 5.
5. Ensure the Light Module and Franka Hand are tightly fixed onto the tool-head.
6. Remove the two M2 screws on the end effector when looking from a top view onto the end effector.
7. Install the Camera Module onto the end effector with the two M2 screws by screwing them through the camera base onto the top of the Franka Hand as seen in Figure 7.
8. To power the light module, connect the USB T7_Connector to the PowerBank.

References

[1] *Academia - Franka Emika*. URL: https://perma.cc/2M5H-T25Z (visited on 03/25/2022).

[2] *Adafruit DotStar*. original-date: 2014-10-09T02:15:45Z. Mar. 27, 2022. URL: https://perma.cc/FE8J-S7AX (visited on 04/10/2022).

[3] *Arduino Proto Shield R3 — 3D CAD Model Library — GrabCAD*. URL: https://perma.cc/R5RA-82CS (visited on 04/03/2022).

[4] *Arduino Uno — 3D CAD Model Library — GrabCAD*. URL: https://perma.cc/4HA7-L3RB (visited on 04/03/2022).

[5] *First Layer Calibration (MINI/MINI+)*. Prusa Knowledgebase. URL: https://help.prusa3d.com/en/article/first-layer-calibration-mini-mini_229122 (visited on 04/01/2022).

[6] Joshua Frank. *ROS Serial Arduino library*. original-date: 2017-04-30T00:41:05Z. Mar. 27, 2022. URL: https://github.com/frankjoshua/rosserial_arduino_lib (visited on 04/10/2022).

[7] *Franka Emika Panda STEP file — CAD file — Download*. WiredWorkers. URL: https://wiredworkers.io/nl/download-franka-emika-panda-step-file/ (visited on 03/30/2022).

[8] *Mallory PB-09N23-03Q Buzzer — 3D CAD Model Library — GrabCAD*. URL: https://perma.cc/85H5-HR5W (visited on 05/02/2022).

[9] *Screw Terminal Block 3.5mm — 3D CAD Model Library — GrabCAD*. URL: https://grabcad.com/library/screw-terminal-block-3-5mm-1 (visited on 04/03/2022).

[10] Paul Stoffregen. *PaulStoffregen/SPI*. original-date: 2014-07-18T18:31:05Z. Mar. 24, 2022. URL: https://perma.cc/S7XZ-GNRA (visited on 04/10/2022).

[11] *Transistor BC547 — 3D CAD Model Library — GrabCAD*. URL: https://perma.cc/PW4R-D6CN (visited on 05/02/2022).

[12] *USB Connector — 3D CAD Model Library — GrabCAD*. URL: https://perma.cc/FCR3-BY3M (visited on 04/03/2022).

[13] *WiFiNINA library for Arduino*. original-date: 2018-07-11T14:30:09Z. Mar. 28, 2022. URL: https://perma.cc/G7PN-RDEE (visited on 04/10/2022).
