# Supplementary material for: Antropo: An open-source platform to increase the anthropomorphism of the Franka Emika collaborative robot arm
Source: PLoS One. 2023 Oct 18;18(10):e0292078. doi: 10.1371/journal.pone.0292078 (PMC10584189; doi:10.1371/journal.pone.0292078)
Supplement: Attachment — Submitted filename: Review Comments PONE-D-23-11948.docx [file pone.0292078.s003.docx]

# Reviewer comments

## Summary

In this paper, the Antropo platform's open-source features are explained, which enable researchers and industry professionals to design anthropomorphic characteristics for cobots. By integrating cameras, light, and sound, the authors propose that cobots are better received by their users and enhance collaborations.

## Strengths of the paper

- accessibility of the platform
- flexibility the system provides for both research and industry
- applicability in both industry and research

## Suggestions for improvement

- Introduction: The introduction could be improved by adding a dedicated statement about the research aim and related work. Lots of work has been done in the social robotics field in regards to anthropomorphism, and the paper could benefit from adding some work from that field into this paper to make your arguments for specific features stronger, as now it seems a bit 'randomly' chosen.
- Materials: I wonder whether the placement of the features is not limiting the robot’s task performance or whether the system will not get damaged too easily as the gripper is the most used part of this robot. Perhaps it would be good to address this.

Furthermore, the choices for specific colors to indicate specific actions might not be as	intuitive because certain colors also have an emotional connotation e.g. red being - angry	and blue – sad. There is a paper by Johnson, D.O., Cuijpers, R.H., van der Pol, D.: Imitating	human emotions with artificial facial expressions. International Journal of Social Robotics 5,	503–513 (2013) That looked at the meaning of different colored eyes for robots and how	they are perceived. Perhaps that can help you to strengthen and or motivate your choices for	colors of the eyes

- Results: it would be nice to implement some user evaluations of your setup to see how it is perceived to add more weight to the accuracy of the proposed actions. Even if it is just something very short as some interview of an exploratory survey.
